# Enabling pan-repository reanalysis for big data science of public metabolomics data

**DOI:** 10.1038/s41467-025-60067-y

**Published:** 2025-05-24

**Authors:** Yasin El Abiead, Michael Strobel, Thomas Payne, Eoin Fahy, Claire O’Donovan, Shankar Subramamiam, Juan Antonio Vizcaíno, Ozgur Yurekten, Victoria Deleray, Simone Zuffa, Shipei Xing, Helena Mannochio-Russo, Ipsita Mohanty, Haoqi Nina Zhao, Andres M. Caraballo-Rodriguez, Paulo Wender P. Gomes, Nicole E. Avalon, Trent R. Northen, Benjamin P. Bowen, Katherine B. Louie, Pieter C. Dorrestein, Mingxun Wang

**Affiliations:** 1 Skaggs School of Pharmacy and Pharmaceutical Sciences, University of California San Diego, La Jolla, CA USA; 2 Department of Computer Science and Engineering, University of California Riverside, Riverside, CA USA; 3 European Molecular Biology Laboratory, European Bioinformatics Institute (EMBL-EBI), Wellcome Genome Campus, Hinxton, Cambridge, CB10 1SD UK; 4 Department of Bioengineering, and San Diego Supercomputer Center, University of California, San Diego, 9500 Gilman Drive, La Jolla, CA 92093-0505 USA; 5 Faculty of Chemistry, Federal University of Pará, Belém, PA Brazil; 6 Center for Marine Biotechnology and Biomedicine, Scripps Institution of Oceanography, University of California San Diego, 9500 Gilman Drive, La Jolla, CA 92093 USA; 7 Department of Pharmaceutical Sciences, University of California, Irvine, California 92697 Irvine, USA; 8 Environmental Genomics and Systems Biology Division, Lawrence Berkeley National Lab, Berkeley, CA 94720 USA; 9 The DOE Joint Genome Institute, Lawrence Berkeley National Laboratory, Berkeley, CA 94720 USA; 10 Collaborative Mass Spectrometry Innovation Center, Skaggs School of Pharmacy and Pharmaceutical Sciences, University of California San Diego, La Jolla, CA USA; 11 Department of Pharmacology, University of California San Diego, La Jolla, CA 92093 USA; 12 Center for Microbiome Innovation, University of California San Diego, La Jolla, CA 92093 USA

**Keywords:** Computational platforms and environments, Mass spectrometry

## Abstract

Public untargeted metabolomics data is a growing resource for metabolite and phenotype discovery; however, accessing and utilizing these data across repositories pose significant challenges. Therefore, here we develop pan-repository universal identifiers and harmonized cross-repository metadata. This ecosystem facilitates discovery by integrating diverse data sources from public repositories including MetaboLights, Metabolomics Workbench, and GNPS/MassIVE. Our approach simplified data handling and unlocks previously inaccessible reanalysis workflows, fostering unmatched research opportunities.

## Abstract (web summary)

Public untargeted metabolomics data hold great promise for discovery but are difficult to access across repositories. Here, the authors develop universal identifiers and harmonized metadata to integrate major databases, enabling streamlined analysis and expanded research possibilities.

## Introduction

The reuse of public data is undergoing a pivotal shift in the metabolomics field, transitioning from single-study analysis to the realm of big data science. This evolution encompasses multiple studies and entire repositories, ideally spanning across diverse repositories. The emergence of public data repositories like MetaboLights^1^ (MTBLS), Metabolomics Workbench’s National Metabolomics Data Repository (NMDR)^2^, and GNPS/MassIVE^3^, coupled with journal and funding mandates to make data publicly available, has fueled the growth of metabolomics data availability over the past decade.

Traditionally, data reuse efforts have focused on individual or a few well-known datasets. However, recent approaches leveraging entire metabolomics repositories have yielded significant biological discoveries. For instance, advanced data searching tools such as the Mass Spectrometry Search Tool (MASST)^4^ and Mass Spectrometry Query Language (MassQL)^5^ have enabled the expansion of bile acid diversity from hundreds to thousands and *N*-acyl lipids from tens to hundreds through the use of the public GNPS/MassIVE repository^6–9^. These endeavors have linked discoveries to microbial producers, organ distributions, interventions, such as antibiotics or dietary changes, and their associations with different health conditions. Additionally, the indexing and metadata curation efforts at GNPS have provided the foundation for projects such as microbeMASST^10^, plantMASST^11^, foodMASST^12^, and tissueMASST^9^ which enable linking known and unknown microbial plant, food and tissue metabolites to putative producers and distributions, respectively.

These proof-of-principle projects have unveiled the potential of data mining at the repository scale. However, each project demanded extensive efforts to collect and harmonize metadata, index file paths, convert data formats, and integrate multiple pre-existing data processing pipelines. Naturally, these tasks necessitated combined expertise in computational metabolomics and software engineering. Moreover, the technical and computational infrastructures required to enable repository scale analyses present substantial barriers. Combined, these challenges become more pronounced when integrating data from multiple repositories.

The repositories MTBLS, NMDR, and GNPS/MassIVE made different design choices for metadata. Metadata for MTBLS is standardized to the ‘Investigation’, ‘Study’, and ‘Assay’ (ISA) model, similar to Sequence Read Archive (SRA) for sequencing, with controlled vocabularies/ontologies annotations where possible. NMDR based their metadata workflow on the Metabolomics Standard Initiative recommendations^13^ with a text-based format called ‘mwTab’ which is similar in principle to UniProt^14^. GNPS metadata submissions are optional and encourage the use of the controlled vocabulary defined in the ReDU framework^15^. This heterogeneity of metadata makes the discovery of relevant raw data across repositories difficult.

In this work, to overcome these obstacles and align with the FAIR (Findable, Accessible, Interoperable, and Reusable) data principles^16^, we present Pan-ReDU, as a major update to ReDU^15^ and a set of tools in the Pan-ReDU computational ecosystem.

## Results

**Fig. 1:**
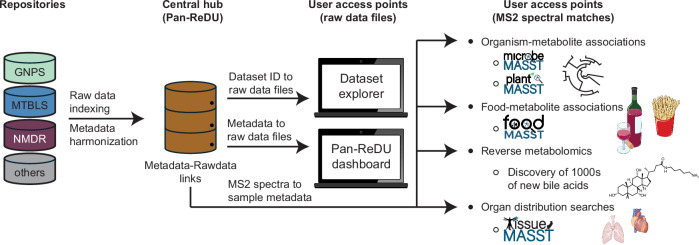
Pan-ReDU workflow. Public metadata are harmonized and formatted into a table with controlled vocabularies/ontologies. Each data file is associated with a unique MS Run Identifier (MRI), which can be used to download data from a selection of more than 600 K raw files or transfer them to different online or offline services. Users can find data files via metadata categories (e.g., Homo sapiens, and blood plasma) or dataset IDs. Fragmentation spectra (MS/MS or MS2) can be searched against public data^10,12,32^ to retrieve metadata categories with matching scans. This work lays the foundation for making this information available to all public repositories. Notably, no programming skills are required for standard users.

**Fig. 2:**
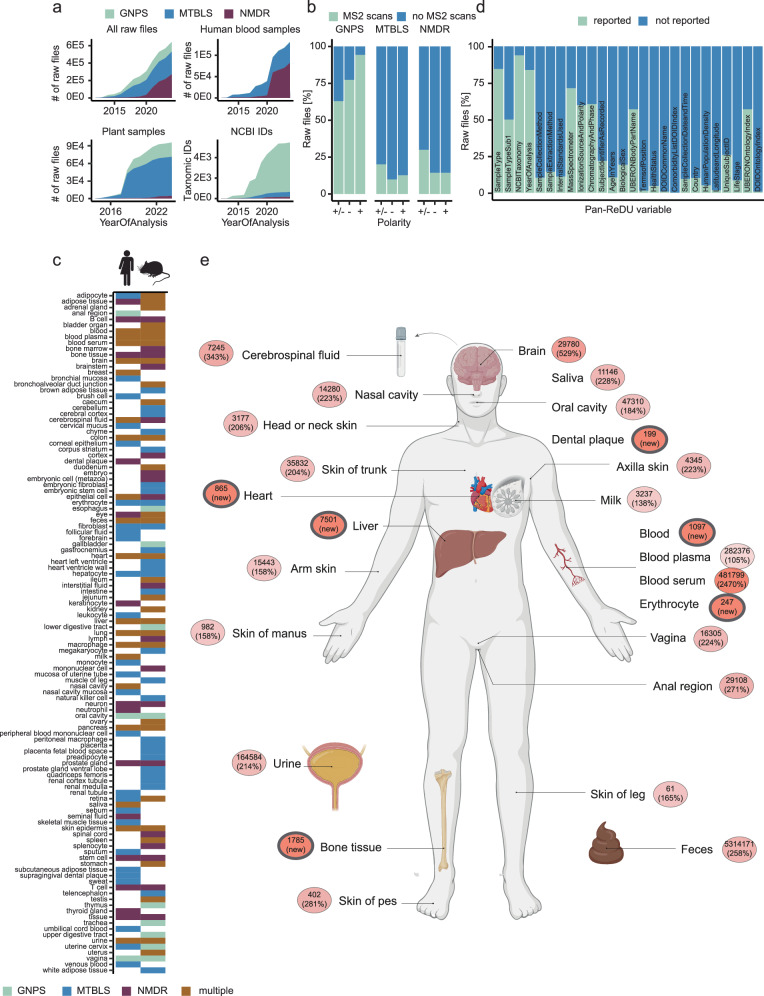
Overview of the harmonized data indexed in Pan-ReDU. The distribution of Pan-ReDU compatible publicly accessible raw files and metadata from MTBLS, NMDR, and GNPS/MassIVE. **a** While MTBLS (blue) and NMDR (purple) have more human blood samples deposited in comparison to GNPS (green), the picture inverts when it comes to the number of unique taxonomic IDs. Additionally, a small number of plant samples have been deposited in NMDR compared to the other two repositories. **b** GNPS contains a higher percentage of MS files for which MS/MS scans were acquired (green), at different polarities, compared to the other repositories, highlighting different study design approaches in the community. MTBLS and NMDR contain a lower percentage as MS/MS scans are often acquired only from a pooled sample. **c** The distribution of analyzed body parts/cell types in Homo sapiens and Mus musculus underlines how the different repositories complement each other. GNPS (green), MTBLS (blue), NMDR (purple), and multiple (orange) are given as color schemes. **d** Proportion of reported Pan-ReDU metadata variables. Reported is given in green, and not reported in blue. **e** Updated numbers of a human body distribution search of bile acids from the recently published Candidate Bile Acid Library utilizing Pan-ReDU. The percentages and color scheme show the increase relative to before the Pan-ReDU effort^7^: the darker the color, the higher the increase. On average the increase is 246% and six new tissues/biofluids (circled in gray) were added. **e** was created using BioRender.com. Source data are provided as a Source Data file.

Pan-ReDU is a computational infrastructure and set of tools that aims to first, harmonize sample metadata into controlled vocabularies/ontologies^15^ and index raw mass spectrometry data files across multiple metabolomics data repositories (MTBLS, NMDR, and GNPS/MassIVE). Second, it aims to facilitate mass spectral raw data access and reanalysis with existing computational pipelines. The third objective is to enhance data download and transparency across multiple repositories by utilizing MS Run Identifiers (MRI)—a subset of the Universal Spectrum Identifiers (USI)^17^. Fourth, to increase data reuse by indexing fragmentation mass spectra (MS/MS) in raw mass spectrometry data to enable MS/MS searches via MASST throughout all supported repositories (Fig. 1). Finally, all data and metadata are integrated into the Pan-ReDU search engine which is the web data portal for the community to find data, download data, and/or co-analyze data across multiple data repositories. Overall, the Pan-ReDU effort has grown the number of data files in ReDU-compatible harmonized metadata format from 38,305 to 644,008 (15 fold increase, Fig. 2a).

To aggregate metadata, all supported metabolomics raw data formats (mzML, mzXML, .raw, .wiff, .cdf, and .d) that could be associated with sufficient metadata [meaning that at least one piece of sample-specific metadata was available (e.g., analyzed organism or body part)] were added to Pan-ReDU. As of April 2024, MTBLS made up 39% of Pan-ReDU compatible raw data, representing ~95% of MTBLS supported raw data; NMDR made up 42% representing ~67% of raw data in NMDR; and GNPS contributed 19% representing ~12% of all GNPS raw data that had Pan-ReDU compatible metadata. Although the three repositories have some overlap between the communities they serve, they do reach different audiences with different preferences regarding sample types and data acquisition modes (Fig. 2a). NMDR, funded by the National Institutes of Health (NIH), supports data deposition of clinical studies and contributed the majority (73%) of human plasma/blood data to Pan-ReDU. This data is often collected without MS/MS spectra for untargeted discoveries (Fig. 2b). MTBLS showed similar proportions of raw data regarding MS/MS spectra and is a global general-purpose metabolomics repository, while GNPS/MassIVE serves a worldwide community that uses mainly untargeted MS/MS-based metabolomics (Fig. 2b), often to discover novel natural products and microbiome-derived molecules. This is reflected in GNPS/MassIVE having the largest diversity of represented organisms (Fig. 2a). Overall, these three repositories complement each other with diverse sample types as well as analyzed body parts within the same organisms (Fig. 2a–c).

As a prime example of the increased coverage of Pan-ReDU, we have repeated a recently published bile acid human organ distribution analysis in Fig. 2e^7^. Using Pan-ReDU metadata, the number of matched bile acids increased by an average of 246% across all organs. Supplementary Fig. 1 shows the increase resolved by the number of hydroxyl groups on the bile acid. Moreover, MS/MS matches for six additional tissues/biofluids not present in previous versions of ReDU were retrieved. Another example workflow showcasing how to retrieve library annotations, for a given sample selection based on Pan-ReDU metadata, is provided in Supplementary Information 1. While abundance based feature analysis across multiple datasets are currently not possible, Pan-ReDU allows users to find and download any dataset of interest—e.g. datasets on inflammatory bowel disease (IBD)—and reanalyze with quantitative in-house feature extraction tools.

Overall, Pan-ReDU makes metadata shareable, searchable, and filterable via interactive web interfaces, simplifying the selection of relevant subsets of public data. Some metadata information such as taxonomy, sample type, and year of analysis are more commonly captured by the metabolomics community, while more granular information such as longitude and latitude, human population density, and build environment are less frequently documented, as less of these data is publicly deposited (Fig. 2d). This can be explained in part by the relative differences in research community sizes. Furthermore, Pan-ReDU improves accessibility by integrating computational tools that streamline the diverse download mechanisms of the different repositories. Additionally, it enhances interoperability by converting raw MS data to the open mzML format^18^. Finally, Pan-ReDU converts metadata information to formats supported by downstream analysis tools like GNPS Molecular Networking and the Sample and Data Relationship Format (SDRF)^19^. By utilizing publicly controlled vocabularies/ontologies, the reusability of metadata terms is ensured, enabling Pan-ReDU to adapt to evolving scientific terminology and reanalysis needs. This holistic approach aims to streamline the handling of public metabolomics data.

The Pan-ReDU raw data retrieval infrastructure builds on MRIs which resolve the location of datasets and the files within. Thus, MRIs and Pan-ReDU provide a standard mechanism for a virtual path to any spectral raw file (in a supported format) in any project contained in the supported repositories integrating respective metadata. We have developed two distinct entry points for interactive use: First, the *Dataset Explorer* enables the selection of files within a specific dataset. We regularly update our index to keep this up-to-date with the latest submitted datasets. Further, metadata within each dataset, when available, can be displayed to enhance the selection of relevant subsets of samples for reanalysis. Second, the *Pan-ReDU* dashboard enables the selection of files across all repositories and datasets by metadata, for example, users could search for “9606|Homo sapiens” and “urine” and receive a list of MRIs for the selected data from all three indexed repositories. These MRIs can be used to download data using the Pan-ReDU resolver tool, thereby enabling the integration of any MS software into local analysis pipelines that support community standards for data and metadata^18^.

To further lower the barrier of entry, MRIs via these interfaces are fully integrated into the actively developed and web-accessible GNPS2 ecosystem. For example, MRIs can visualize and inspect raw mass spectrometry without any user downloads using the GNPS Dashboard (Supplementary Table 1). Further, Classical Molecular Networking within the GNPS2 cloud platform (also available as a standalone NextFlow workflow) natively supports MRI downloads by integrating with the Pan-ReDU MRI resolver—enabling reanalysis without users having to manually download and upload files. This workflow is publicly released as open source (UC ShareAlike License) and can also be run by any users on their local system with the same integration to the existing public repository data.

Finally, we have created a standalone software package (*publicdatadownloader*) that allows users to easily download any set of valid MRIs from the supported repositories. While we have demonstrated direct use of *publicdatadownloader* in the Classical Molecular Networking workflow, as a Python-based command line tool, integration into any other workflow can be achieved.

Even with several prominent publications demonstrating the discovery potential of re-analyzing public data^6,7,10,20^, many in the metabolomics community currently do not understand the value of depositing data in the public domain. Additionally, comparing the number of published MS-based metabolomics articles (11,187 in the last 5 years, according to a Web of Science search—see “Methods”) to the public datasets uploaded to the repositories during the same time (2013 datasets in Pan-ReDU), it is evident that the majority of data remains unshared. However, there is a growing cohort of metabolomics researchers embracing data sharing and reuse. Over the last 7–8 years, 518 TB were downloaded from GNPS, 192 TB from MTBLS, and ~30 TB from NMDR. Pan-ReDU aims to bolster the value of public data deposition by enhancing reusability and impact. Furthermore, these endeavors lay the groundwork for future developments, enabling the creation of tools for more efficient and intuitive public data utilization by the metabolomics community from all compatible metabolomics repositories, including the ones that will emerge in the future. By reshaping these incentives, we anticipate a shift in community standards and expectations toward increased data sharing and deposition.

## Methods

We have implemented a series of Python 3.8.8 command line tools within Nextflow (version 23.10.1.5891) workflows as described in the sections below.

### Indexing and accessibility of raw data

We have employed a systematic approach to index raw metabolomics data across a diverse range of repositories, namely GNPS/MassIVE, NMDR, and MTBLS, by leveraging the capabilities of their API tools. To facilitate easy retrieval, each supported mass spectrometry file (.raw, .d, .wiff, .cdf, mzML, and mzXML, all of them are called collectively raw data) is indexed across all of these public resources and referenced by the MS Run Identifiers (MRI). To achieve this, we have utilized MRIs, a subset of the USI standard, to incorporate not only scans but also the dataset IDs and the file path, serving as a comprehensive unique identifier that enables access to all aforementioned repositories from the GNPS2 ecosystem. The service to translate MRIs to file paths is provided at https://dashboard.gnps2.org/. Ultimately, MRIs serve as a handle for the download and processing of MS files from any repository via a software package that has been implemented in multiple tools in the GNPS2 ecosystem including MassQL and Classical Molecular Networking. Notably, we developed tooling to convert the main proprietary MS file formats (.cdf, .raw, .wiff, and .d) into the open MS format mzML^21,22^. Files can be downloaded by users after conversion to mzML or as vendor format.

### Retrieval of raw data via MRIs

By providing a set of MRIs researchers can trigger a three-step process for comprehensive data accessibility.

#### Step 1—caching

The workflow efficiently utilizes an internal cache, storing previously downloaded datasets. If the requested data resides within the cache and passes integrity verification (i.e., .mzML have to be valid XML files), it is immediately available, minimizing wait times and resource consumption. For vendor files, data is converted using the GNPS2 Dataset Cache and converted versions can be requested and made available for download for 1 month.

#### Step 2—download

In the absence of locally cached data or if the integrity is compromised, the workflow initiates download from the corresponding repository. This ensures researchers access the most recent data, regardless of its location.

#### Step 3—transparent reporting

For optimal transparency, the workflow generates detailed reports for each MRI. These reports provide insights into download success rates, cache utilization, and any encountered errors, enabling researchers to monitor progress, identify potential issues, and ensure data integrity for robust analysis.

By integrating user-friendly functionality with intelligent caching, automated downloads, and transparent reporting, our workflow streamlines mass spectrometry data retrieval.

### Harmonization of metadata

ReDU metadata is organized in a single table for all available datasets and it relies on controlled vocabulary. NMDR’s mwTAB format^23^ uses a key-value data structure for each individual study, and MTBLS leverages the ISA framework^24^ with multiple tables (for the sample, assay, and metabolite level) for each deposited study, comprising a combination of controlled ontologies and free text.

To bridge this gap, we developed a Python workflow for automated metadata integration from NMDR and MTBLS to ReDU. Only raw files with at least one piece of information on the nature of the sample (e.g., analyzed organism, or solvent blank) were accepted into the Pan-ReDU table accessible via https://redu.gnps2.org/.

### Pan-ReDU Metadata Selection Dashboard

The Metadata Selection Dashboard was built as a Dash app. Automated updating to the latest metadata uploads from users was built in to be activated daily. Linkouts to GNPS2 Molecular Networking, MassQL searches, and in-browser raw data inspection through GNPS2 Dashboard have been built into the dashboard for easy access.

### Pan-ReDU Metadata Validation Sheet

To validate metadata before submitting it to Pan-ReDU, a Google Sheet utilizing Google Apps Script, written in JavaScript, was developed for easy and intuitive access. Different sub-sheets with documentation, explanations of metadata terms, allowed terms, and a demonstrative example of how to validate the metadata were provided. New terms and Pan-ReDU categories will be updated in this sheet (see in “Data availability”).

### Pan-ReDU Metadata Submission System

An updated submission system for Pan-ReDU metadata was developed as a Dash app. It was set up so users can submit metadata for their own datasets after providing login credentials for GNPS/MassIVE. Metadata are then deposited into a GitHub repository from which metadata will be pulled daily into Pan-ReDU.

### Workbench’s National Metabolomics Data Repository

For every individual study accession, raw data file names were sourced via the NMDR’s API. We relied on NMDRs REST service to gain information specifying the type of each sample (e.g., T-cells or Sweat) in the form of an internally controlled vocabulary. Further metadata were sourced as mwTAB files and parsed according to the specifications provided in the mwTab documentation. mwTab provides predefined slots for each NMDR-controlled vocabulary entry or free text entry of variables (e.g., SUBJECT_SPECIES, CHROMATOGRAPHY_TYPE, and COLUMN_NAME) on a per-analysis basis. We relied on combinations of substring matching and translation sheets to convert all terms into the ontology used in Pan-ReDU.

Translation sheets were created in part manually after inspection of terms used in NMDR and rely in part on public ontologies defining commonly used terms (including synonyms) for entities. Specifically, we incorporated ontologies of body parts^25,26^, plant organism parts^27^, cell types^28^, disease names^29^, and MS technical information including mass spectrometers^30^.

Raw data file names were read from the REST-service provided table if available. Otherwise, we inspected the “Additional sample data”, “Factors” or “Sample ID” sections for information on provided raw files. We attempted to associate deposited spectral raw data with file names in the user-provided metadata by using full paths, base file names, and base file names without extensions. If none of these matched, we also tested whether multiple spectral file names have been deposited as a single value by splitting files via the different separators (“,”, “;”, or “”). Finally, if none of these approaches led to matches, we attempted substring matching between the base file names of the actual spectral raw files and the descriptors provided by uploaders. If unambiguous matches were found, they were accepted. Studies where we could not associate any spectral data to specific samples were discarded.

In cases where multiple mwTab files were deposited for the same study, it was not possible to retrieve which raw file belonged to which mwTab file. Therefore we attempted to associate raw files to mwTab files by matching the polarity mode reported in the mwTab files, to the polarity reported in the scan headers of the respective mzML/mzXML files. This allowed us to rule out certain spectral file-mwTab file associations. If the association to a specific mwTab could not be established ambiguously, metadata was not considered.

### MetaboLights

For each study identifier, file paths and metadata were requested via the MetaboLights API. Afterwards, equivalent metadata columns were matched between MTBLS and ReDU. For example, “NCBITaxonomy” was matched to “Organism”, “UBERONBodyPartName” to “Organism part”, “Instrument” to “MassSpectrometer” and so on. MTBLS already uses publicly controlled vocabularies/ontologies allowing easy matching to the ReDU ontology. For ReDU columns where no equivalent predefined column was present in MTBLS, we attempted to find the equivalent columns via the non-default column names the submitter employed. For example, to retrieve the ReDU variable “BiologicalSex” we looked at whether a factor column “sex” or “gender” was deposited. If the same MS raw file was available in multiple formats, only the open format (mzML or mzXML) was kept.

### Scan type overview

For ReDU and NMDR, scan type statistics were computed after downloading mzML and mzXML files from the respective repositories and extracting scan levels and polarities contained in the individual raw files. The statistics are based on raw files downloaded via the respective repository APIs. In the case of MTBLS, statistics are based on polarity scan level information that was provided by the MTBLS team.

### Extraction of scan counts from raw files

Scan counts have been retrieved directly from the MS raw data (mzML and mzXML files) using pyteomics^31^ (version 4.7.2) after downloading MS raw data through the Publicdatadownloader developed here. Downloaded raw data are set to be updated to keep adding scan counts to the newly deposited raw data.

### Assessing research article to data sharing ratio

MS-based metabolomics articles were searched using Web of Science via the keywords “metabolomics” and “mass spectrometry” in any field only considering Research Articles published between 01/01/2019 and 01/01/2024. The number of shared public datasets in Pan-ReDU was assessed by considering Pan-ReDU datasets with a YearOfAnalysis in 2023 or earlier.

### Reporting summary

Further information on research design is available in the Nature Portfolio Reporting Summary linked to this article.

## Supplementary information

Supplementary Information

Reporting Summary

Transparent Peer Review file

## Source data

Source Data

## Data Availability

Dataset Explorer—Web user interface for public data search by Dataset accession ID https://explorer.gnps2.org/. ReDU Pan-Metadata Dashboard—Web user interface for public data search by Metadata category https://redu.gnps2.org/. Pan-ReDU Metadata Validation Sheet https://docs.google.com/spreadsheets/d/10U0xnJUKa_mD0H_9suH1KJAlJD9io9e4chBX8EAHneE/edit?usp=sharing. Pan-ReDU Metadata Submission System https://deposit.redu.gnps2.org/. Pan-ReDU documentation https://wang-bioinformatics-lab.github.io/GNPS2_Documentation/ReDU_overview/https://wang-bioinformatics-lab.github.io/GNPS2_Documentation/downloadpublicdata/. Pan-ReDU Metadata Storage https://github.com/Wang-Bioinformatics-Lab/ReDU_metadata. Pan-Repository Full MRI Index https://datasetcache.gnps2.org/datasette/database/filename. Raw File Visualization Dashboard—Web user interface https://dashboard.gnps2.org/. Source data are provided with this paper.

## Code availability

ReDU—source code. https://github.com/Wang-Bioinformatics-Lab/PublicDataset_ReDU_Metadata_Workflowhttps://github.com/Wang-Bioinformatics-Lab/ReDU-MS2-GNPS2https://github.com/Wang-Bioinformatics-Lab/GNPS_DatasetExplorer. Classical Molecular Networking NextFlow Workflow—source code https://github.com/Wang-Bioinformatics-Lab/Classical_Networking_Workflow. Publicdatadownloader—command line download utility https://github.com/Wang-Bioinformatics-Lab/downloadpublicdata.
