# Supplementary material for: Enabling pan-repository reanalysis for big data science of public metabolomics data
Source: Nat Commun. 2025 May 24;16:4838. doi: 10.1038/s41467-025-60067-y (PMC12103507; doi:10.1038/s41467-025-60067-y)
Supplement: Supplementary file 1 — Supplementary Information [file 41467_2025_60067_MOESM1_ESM.pdf]

Supplementary Information

Supplementary Figure 1

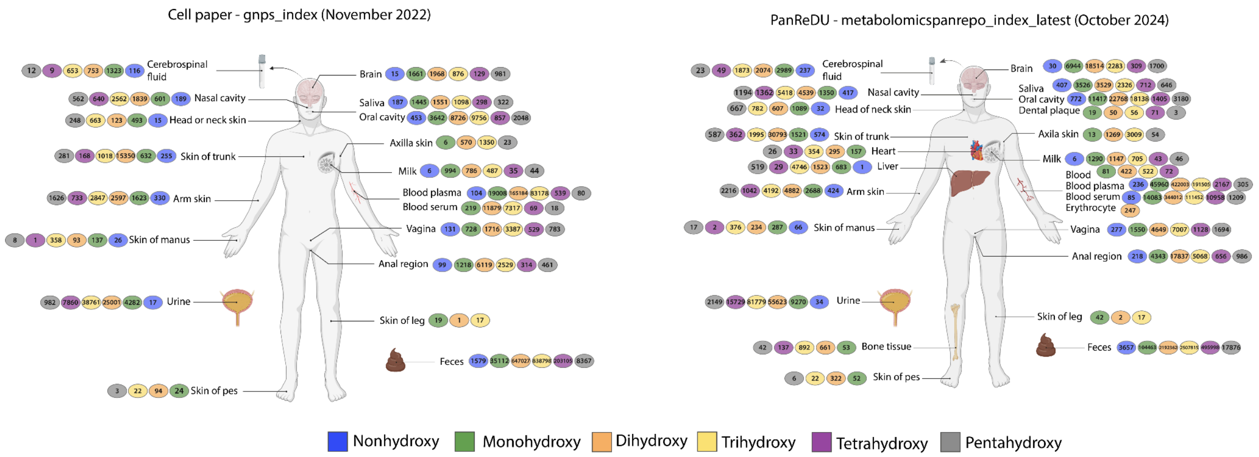

Supplementary Figure 1. Bile acid matches the number of hydroxy groups on the bile acid core. Updated numbers of a human organ distribution search of bile acids from a recently published Candidate Bile Acid Library utilizing Pan-ReDU. The left panel shows the numbers before the update, and the right panel shows the numbers after the Pan-ReDU update[7]. On average, the increase was 246% per tissue/biofluid type. The numbers are given separately by the number of hydroxyl groups on the bile acid core. This figure was made using BioRender.com. Source data are provided as a Source Data file.

Supplementary Table 1

Supplementary Table 1 | Linkouts demonstrating the accessibility of different file formats across different repositories within the browser

MS Run Identifier (MRI)
mzspec:MSV000094405:MSV000094405/raw/SCCe0064.mzML
mzspec:MTBLS300:FILES/Method 4 stool/QXA07POSLAT20190401_MSST010118ML_HUMAN_FECAL4_08.mzML
mzspec:MSV000094460:raw/FIR094/20221108_C1_094_R2A_ext_POS_2-B,7_01_35551.d.mzXML
mzspec:MTBLS1039:FILES/DERIVED_FILES/13-124_12hpi_Chitin_1_150306.mzXML
mzspec:MSV000094118:raw/Raw/20210552_QC5.raw
mzspec:MTBLS1024:FILES/KN210_1019_ppl_neg_400k.raw
mzspec:ST002244:Positive_QC_16.raw

Supplementary Note 1

Here we demonstrate how we can select and process *Mus* and *Mus musculus* mzML and mzXML with files with MS/MS scans to retrieve GNPS library annotations and molecular networking results.

1. Go to the ReDU data selection dashboard at https://redu.gnps2.org/selection/
2. Set the NCBITaxonomy column filter to 'contains ".(Mus|Mus musculus)$"' which retrieves all NCBI Taxonomy values ending on 'Mus' or 'Mus musculus'.
3. Set the UBERONBodyPartName filter to '= kidney' which retains all kidney samples.
4. Subset to files that contain MS/MS spectra by setting the MS2spectra_count to '> 0'.
5. Select a few rows via the checkboxes on the left side of the table and click the 'Molecular Networking/Library Matching' button (or download all filtered MRIs to submit more than can be selected on a single page).

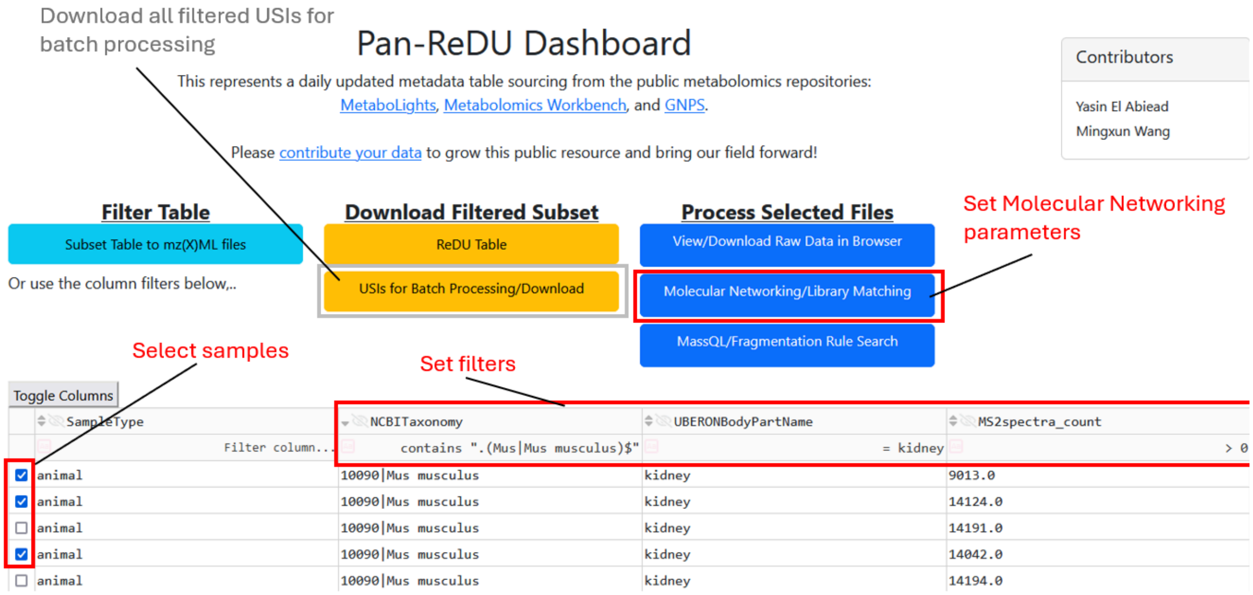

6. This will bring you to the Molecular Networking submission page with the MRIs of the selected files already populated.
7. Give a title to your job, set 'Integrate ReDU Metadata for Public USIs' to 'Yes', and the other parameters to your satisfaction.
8. Submit workflow through the 'Submit Workflow' button.

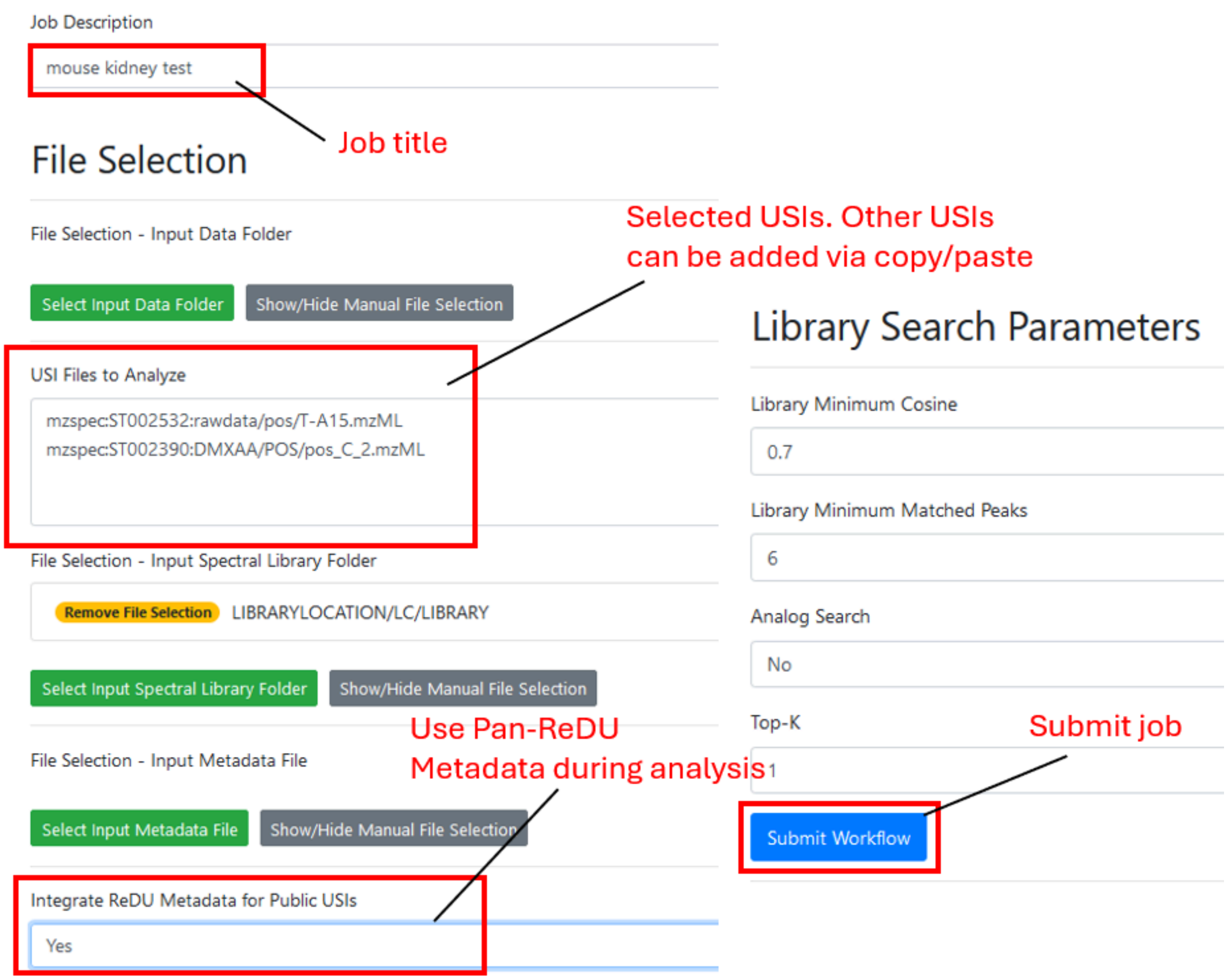

9. Once the status page switches from Status ‘Running’ to ‘Done’ you can download all results via the ‘Download All Results’ button.

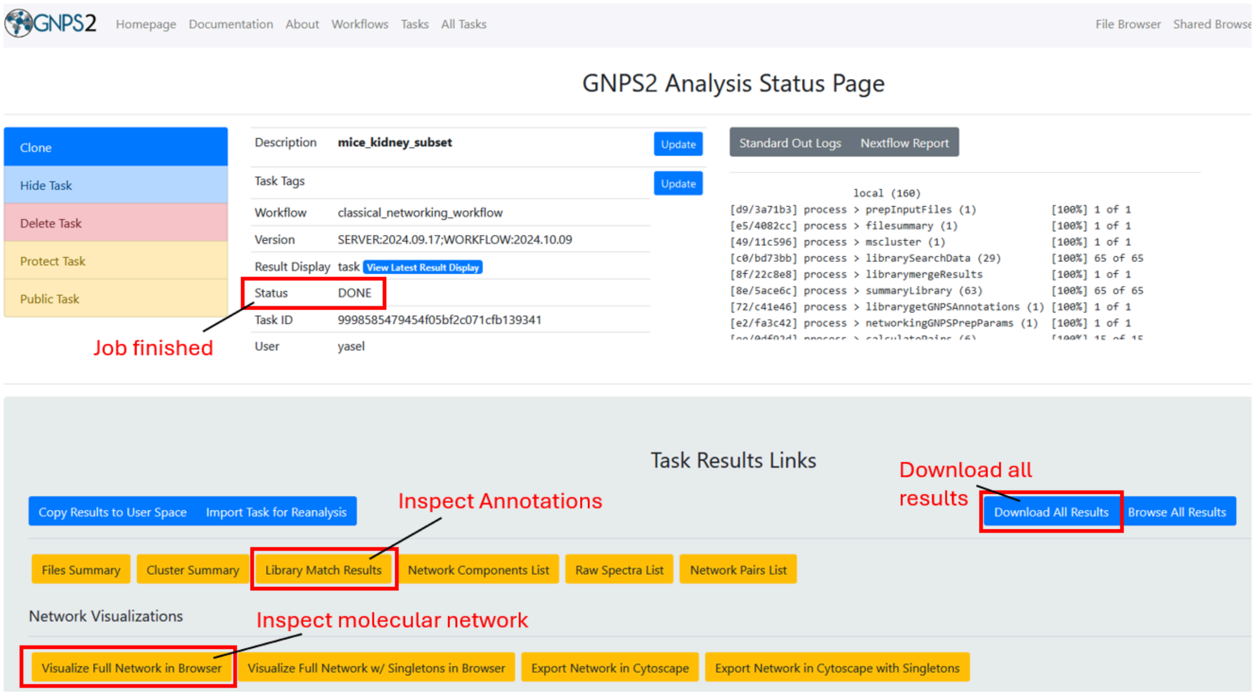

10. Click the ‘Library Match Results’ button to inspect all molecular annotations.

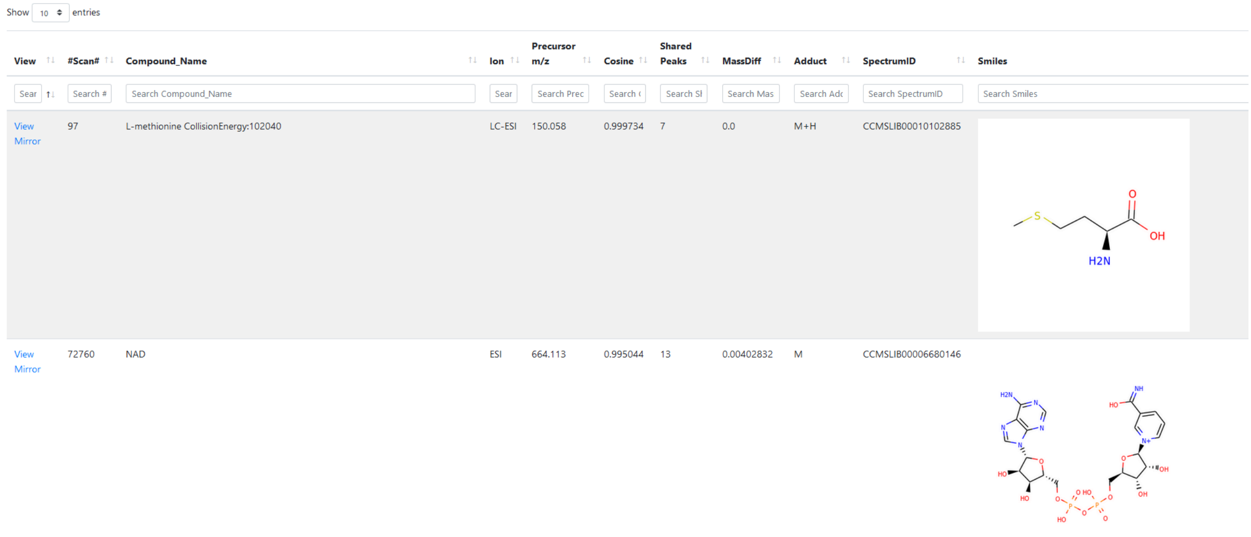

11. You can click the ‘Visualize Full Network in Browser’ button for an interactive Molecular Network.

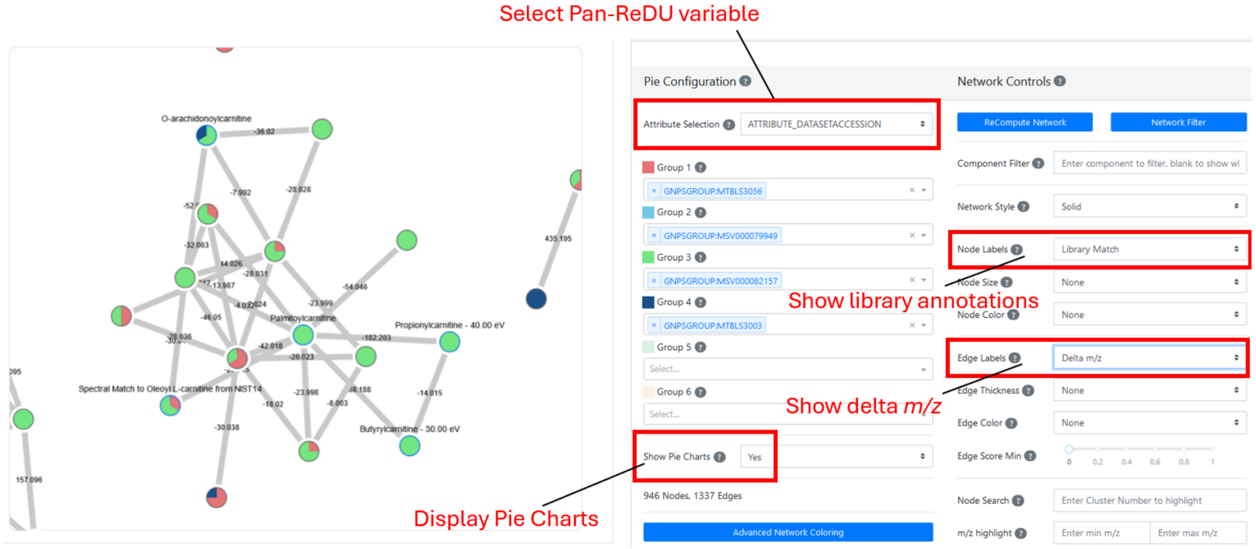

12. The ‘Visualize Full Network in Browser’ button will bring you to an interactive network. It might require some time to load depending on the size.
13. To integrate with metadata set ‘Show Pie Charts’ to ‘Yes’, and select any ReDU column from the ‘Attribute Selection’ dropdown menu. This will show the relative number of observed spectra for every node as a pie chart per selected grouping.
14. To display annotations set ‘Node Labels’ to ‘Library match’. Clicking on individual nodes allows to show structures of molecules if available for the respective annotation, as well as MS2 spectral matches.
